# Evaluation of *Schistosoma Mansoni* Morbidity One Year After Praziquantel Treatment in Rhino Camp and Obongi in West Nile, Uganda

**DOI:** 10.4314/ajid.v4i2.55147

**Published:** 2010

**Authors:** E I Odongo-Aginya, T L Lakwo, Med Ekkehard Doehring

**Affiliations:** 1 Gulu University, Faculty of Medicine, Microbiology Department. P.O.Box 166 Gulu; 2 Vector Control Division P.O.Box 1661 Kampala Uganda; 3 University of the Mountain of the Moon. P.O.Box 834 Fort Portal, Uganda

## Abstract

An evaluation study on reversibility of *Schistosoma mansoni* induced periportal fibrosis (PF) morbidity following treatment with praziquantel, 40mg/kg body weight after one year, was carried out in Rhino Camp and Obongi all are West Nile districts in northern Uganda. To assess the reversibility of *Schistosoma (S) mansoni* induced PFs morbidity following treatment with praziquantel, 40mg/kg body weight after one year. The design was a Prospective cohorts study; and the setting was a busy canoe landing sites along Albert Nile in *Schistosoma (S) mansoni* hyperendemic areas of Rhino Camp and Obongi fishing village were selected for the study. Previously in 2005, 1562 people including fishermen and women, school pupils, teachers, and civil servants were studied in both fishing villages for *S. mansoni* using Kato/Katz stool smear method. Abdominal ultrasonography and sonomorphological abnormalities of periportal fibrosis were performed with Aloka portable ultrasound machine (Hellige, Freiburg, Germany) fitted with a convex probe of 3.5 mega Hertz was also performed in the field clinic on all patients who had *S. mansoni* eggs in their faeces. The sonomorphological abnormalities of periportal fibrosis were categorised and organomorphometry of liver and spleen was done. One thousand two hundred and seventy three 1273 (81.5%) patients in Rhino Camp and Obongi fishing villages were found to be excreting from 100 to ≥ 500 eggs per gram (epg) of faeces of *S. mansoni* eggs. Two hundred and eighty nine (18.5%) did not have eggs of *S.mansoni* in their faeces. All the 1273 patients secreting eggs of S.mansoni in their stool in Rhino Camp and Obongi fishing villages had abdominal ultrasonography and sonomorphological abnormalities of periportal fibrosis. Eight hundred and forty 840 (66%) although excreted *S. mansoni* eggs in their stool had Pf (0); Pf grade (I), n=259 (20.3%); Pf grade (II) n =147 (11.5%); and Pf grade (III) n=27 (2.1%) were observed.

## Introduction

Schistosomiasis mansoni was first described in Uganda in Arua district, in West Nile, the area of the present study in 1902. This was the first time ever *S.mansoni* was observed (Castellani, 1903; Castellani and Low 1904). Since then the Districts of West Nile, the areas for this study drew a lot of attention in the study of clinical, epidemiology and pathology of *S.mansoni* (Nelson 1958; Ongom and Bradley. 1972., Ongom et al., 1997). Nelson was the first to make a complete assessment of the incidence, distribution and importance of schistosomiasis mansoni as a health problem in Uganda especially in West Nile (Nelson, 1958a). He also found that the prevalence and intensity of infection was highest immediately along the bank of the River Nile and decrease with altitude and distance from the bank of the Rive Nile. Nelson further observed that enlarged spleens and anaemia was common clinical feature among young children of ten years old with intense infections with Bilharzia (Nelson, 1958b). In resent studies in Rhino Camp, Adama showed deterioration in the morbidity due to *S.mansoni* infection (dissertation for Health Visitor Collage Kampala-Uganda, 1986). In the same year, Bukenya and Adama reported a prevalence of 37% in Rhino Camp (Bukenya and Adama, 1986).

Previously in Uganda and elsewhere in the tropical countries where *S.mansoni* is endemic, the study on the morbidity of *S. mansoni* infections was based on the intensity of the egg excretion, and clinical methods to describe the pathological sequelae induced by the parasites. These methods included the quantitative estimation of *S.mansoni* eggs in the stool specimens, palpation of liver and spleen, biopsy of liver and kidney, X-ray and autopsy of the liver, kidneys spleen and other internal organs. Any of these methods or in combination as applicable were used to detect the morbidity and pathology due to *S. mansoni*. By these methods it was difficult to accurately assess the degree of pathology in these organs (Nelson 1958a or 1958b//; Ongom et al. 1972, 1997). Beside, the invasive needle biopsy of these organs in inexperience hands was risky and sometimes fatal. This made monitoring of reversibility of pathology in these organs after schistosomal chemotherapy difficult (Ongom et al., 1997). Abdel Wahab introduced a non-invasive ultrasound as a tool to detect pathology due to *S. mansoni*. These pathological lesions are common in most internal organs. The sexually mature female and male worms pair in the liver and move out into the mesenteric veins in the small intestine where they dwell and start to lay eggs. This is true in cases of *S. mansoni, S. japonicum, S. intercalatum*, and *S. mekongi*. Nevertheless, *S. haematobium* move to the wall of the bladder where they reside. One to two months after initial infection, clinical signs and symptoms develop. These symptoms include clinical toxaemic fever, Katayama fever common in schistosomiasis mansoni and schistosomiasis japonicum (Ana et al,. 1995), weakness, weight loss, diarrhoea, abdominal pain, urticaria and marked eosinophilia are clinical symptoms commonly seen in the acute phase of infection that progresses to the chronic phase. After three to five months, the chronic intestinal phase (IN) sets in. During IN stage schistosome eggs are present in stool. This stage is followed by hepatointestinal (HI) stage. In this stage *S. mansoni* eggs increase in stool and the liver becomes palpable (Chen and Mott, 1988). Hepatosplenic (HS) schistosomiasis is the established chronic phase of schistosomiasis usually resulting from heavy *S. mansoni* infection. In the HS phase, the clinical symptoms observed in acute phase are apparent and both the liver and spleen are palpable. This stage is closely associated with periportal fibrosis, portal hypertension, collateral circulation, oesophageal varices, ascites and heamatemesis (Gazzinelli et al,. 1985, 1992; Gerspacher-Lara et al,. 1998). In human schistosomiasis, the eggs laid by female schistosome cause morbidity, which is the severity of the disease (Gryseels and Poldernan, 1991). The circulating eggs can reach nearly all organs like the spleen, kidney, heart and occasionally the brain. These eggs are immunogenic. They elicit immune responses in the host (Amelia et al., 2000) with granuloma formations around the eggs which later become fibrotic and calcified in the venous wall. Portal veins and the surrounding periportal veins are commonly affected.

This non-invasive repeatable method of Abdel Wahab for detecting periportal fibrosis due to *S. mansoni* led to more detailed revelation of pathological feature in internal organs of patients infected with *S. mansoni* and allows the monitoring of the reversibility of the liver periportal fibrosis after schistosomal therapy (Houston et al., 1993). In most *S. mansoni* endemic areas longitudinal observations of reversibility of the periportal fibrosis after treatment with praziquental 40mg/kg body weight are still scarce (Doehring -Schwedtfeger et al., 1992). Study of this kind would help to determine the epidemiology and praziquantel chemotherapy regimen required for control of schitosomiasis morbidity in hyper endemic areas (Homeida et al, .1988; Doerhing Schwerdtfeger et al,. 1990; Abdel- Wahab et al,. 1990).

The aim of this study was to evaluate the effect of treatment with praziquantel, 40mg/kg body weight one year later and the reduction of the eggs excretions, on the reversibility of *S. mansoni* induced liver periportal fibrosis and to observe specific dynamic developments of *S mansoni* induced liver parenchyma alterations among the 1273 patients previously studied in 2005 (Houston et al., 1993; Doehring -Schwedtfeger et al., 1992, Homeida et al., 1988; Doerhing Schwerdtfeger et al., 1990; Abdel- Wahab, et al., 1990).

## Materials and Methods

In 2005, 1562 people including fishermen and women, school pupils, teachers, and civil servants were enrolled and studied in Rhino Camp and Obongi fishing villages in Northern Uganda for *S. mansoni* using Kato/Katz stool smear method. Each village had a population between 4,000 and 6,000 people. Rhino Camp is about 60 Kilometres East of Arua town, and it is located 3^0^ North and 31^0^ East. Here (n=733) 46.9% people were screened microscopically for *S. mansoni* infection. Obongi is located about 3.5^0^ North and 31.5^0^ East. Likewise in Obongi (n=829) 53.1% people were examined microscopically for *S. mansoni* infections. Obongi is about 100 kilometres north of Arua town (Odongo-Aginya et al,. 2002). The main activity in Rhino Camp and Obongi villages is fishing, although, there is low scale cultivation along the Nile where the inhabitants plant food crops like vegetables, cassava and potatoes. There is a poor earth surface road system connecting the two villages used by few lorries which come to these villages on market days. Therefore, the inhabitants of the two villages mainly depend on canoes as their means of transportation.

The stratification of the study groups in the two fishing villages was similar. Firstly, the registered and licensed fishermen and women operating fishing canoes at the landing sites including their families were all included in the study. Secondly, the pupils in primary schools situated about 100 meters to the river were all studied. Thirdly, a small number of civil servants including teachers, police, local administrators, and priests, people with low water contact were also registered in the study. The fourth group included volunteers from the community who came and enrolled in the study every morning.

### Stool examination for parasites

Each patient participating in the study was given a stool container and was asked to return it with about 10 gram of his or her stool the next day. Three slides containing 41.7mg of sieved stool were processed using Kato/Katz method (Katz, et al., 1994). The arithmetic mean egg count of the three slides examined was multiplied by a factor of 24 to obtain the egg count per gram faeces.

### Pathological study using portable ultrasound machine

Ultrasound observers were blinded of the result of all the tests, especially, egg count in both studies in 2005 and 2006, and Pf stages results from the previous year 2005. Ultrasound was performed using the same high quality Aloka portable ultrasound machine (SSD-500, Hellige-Aloka, Freiburg, Germany) in both studies in 2005 and 2006. It was equipped with 3.5 MHZ convex transducer. Photo documentation was done using a Mitsubishi p 66 E-Thermal printers. Abdominal ultrasound was done with the patient lying in dorsal position according to the most recently proposed technique (Jenkins et al., (1992). The sonomorphological abnormalities of periportal fibrosis were categorised according to the Managil score (Cairo working group, 1992). Organomorphometry of liver and spleen was done according to Dittrich et al. (1983) that is, liver length in three different sections, external diameter of extrahepatic portal veins, and three PF branches were measured in millimetres. The grading of the liver fibrosis was done according to Doerhing Schwerdtfeger et al. (1990) as follows: -

**Table:** 

0	No echogenicity with smooth texture around the portal veins. This represents grade (0).
1	Echogenicity with smooth irregular texture around periportal veins and gall bladder neck. This represents grade (I) PF.
2	Broad echogenicity >10mm in the central and around periportal veins indicate grade (II) PF.
3	Involvement of complete liver characterized by echogenicities streaks not confined to periportal veins only. This indicates grade (III) PF.

The same people studied in 2005 who had *S. mansoni* eggs in their stool came back for review in 2006 one year later. Nine hundred and thirty nine n=939 (73.8%) out of n=1273 people were not excreting *S. mansoni* eggs in their stool detected using Kato/Katz method Katz et al. (1994) while 334 (26.2) were still excreting *S.mansoni* in their stool. The intensity of the eggs excreted in 2006 was lower than the previous year. Nevertheless the abdominal ultrasound scan was done on all 1273 again in 2006. The recovery of all the patients studied in previous year has been possible because of the short period, the good mobilisation of the community by the local authorities and the willingness of the patients to participate in the study because of the free treatments and inducement offered to them during the study. Because of this, some of the participants walked long distances to come to be examined and treated. The same procedures used in the previous year were repeated during the re-evaluation study. Detailed clinical investigation was done with the help of the health workers .The clinical questions asked were, information on diarrhoea, blood in stool, haematemesis, and abdominal pains.

### Treatment

Stool for microscopic examinations were all done in the morning hr when the patients had just brought in their stool specimens. Those who were found excreting eggs of *S. mansoni* in their stool were treated with a single oral dose of Praziquantel in the field clinic the next day in the morning by nurses who were trained in the procedures for the treatment. The patients were advised to eat before coming for treatment. Their weights were taken and interpolated against pre-calculated dosages chart containing the number of the 600 mg tablets of Praziquantel (FROM MEDOCHEMIE LTD.LIMASSOL-CYPRUS EUROPE) required to achieve 40 mg/kg body weight. Pregnant mothers were not treated with praziquantel until after birth. Other intestinal Helminths and protozoa were treated accordingly. In addition, ailments which could not be treated in the study clinic were referred to other Health facilities and Arua Hospital.

### Inclusion criteria

- Permanent residents of Rhino Camp and Obongi fishing villages.
- Residents who have fully consented to participate in the study.
- Children between 5 and 18 years old who were granted full permission to participate in the study by their parents/ guardians and who signed the ethical form on behalf of their children.
- Individuals who had not had antischistosomal treatment six months prior the study.

### Exclusion criteria

- Non-residents of Rhino Camp and Obongi fishing villages.
- Residents who declined to participate in the study.
- Individuals who had had treatments with antischistosomal drugs six months before the study.
- Those individual who were very ill with chronic ailments.

### Ethical consideration

This study received clearance from the Uganda Government Ministry of Health ethical committee and approval of Uganda National Council of Science and Technology all based in Kampala Uganda. Informed consent was obtained from the adults but in cases of children under eighteen years of age, their parents' consent was requested. The community was educated on the scope of the study in the local languages and they were asked to decide on the participation of their children.

## Results

**Figure 1:**
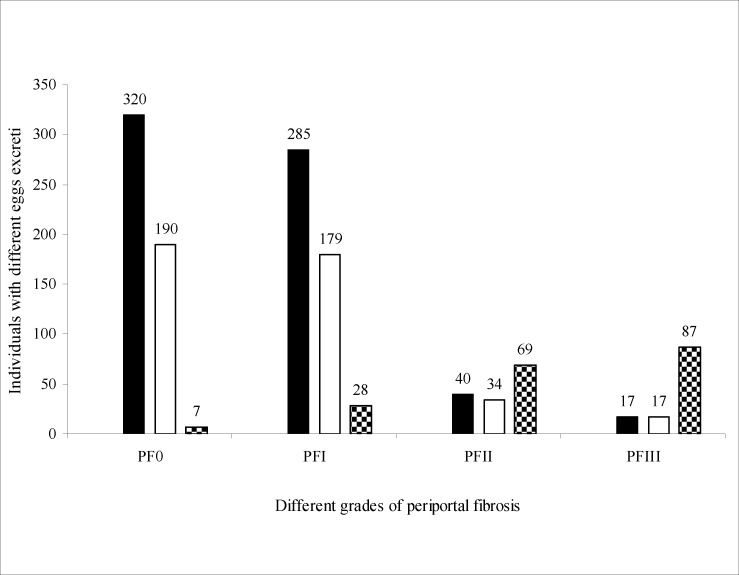
The distribution of periportal fibrosis according to the intensity of infection in 2005 Relationship between different levels of intensity of infections and grades of periportal fibrosis among patients studied in Rhino Camp and Obongi in Northern Uganda, 1–99epg ▪; 100– 499epg □; ≥ 500epg 
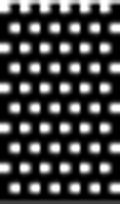

**Figure 2:**
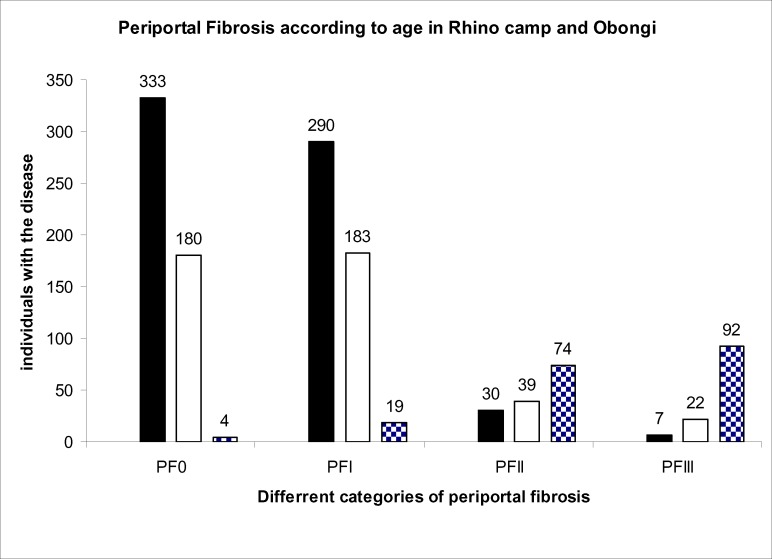
Periportal fibrosis according to age group in Rhino Camp and Obongi in 2005 Periportal fibrosis in different age groups in years in Rhino Camp and Obongi in Northern Uganda 1–10 ▪; 11–20 □; ≥30 
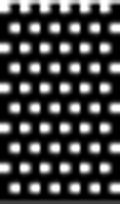

**Figure 3:**
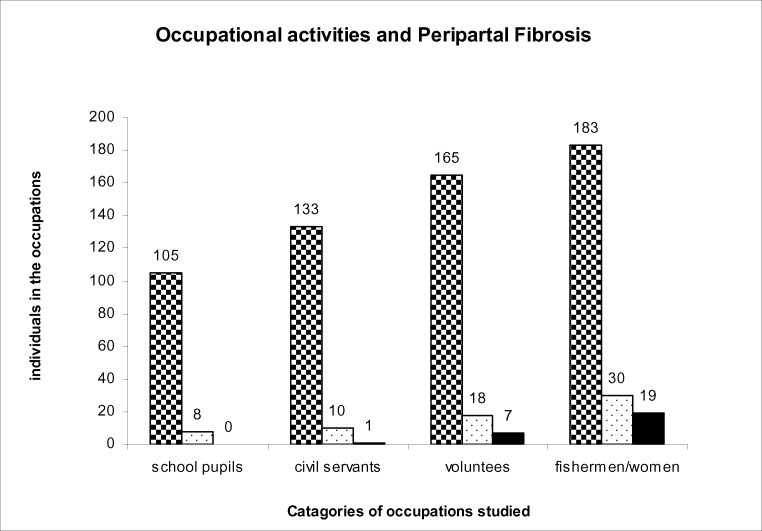
showing relative influence of occupational activities of Periportal Fibrosis in Rhino Camp and Obongi 2005. Periportal fibrosis in different categories of patients studied at Rhino Camp and Obongi in Northern Uganda according to their occupations; Pfs I 
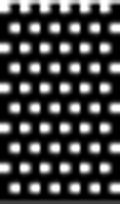
; Pfs II □; Pfs III ▪

**Figure 4:**
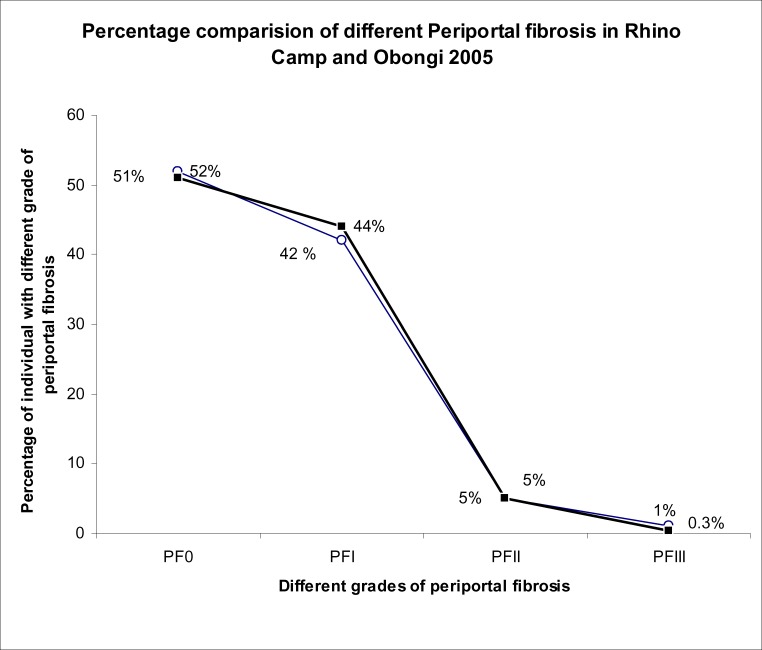
Comparing different grades of periportal fibrosis in Rhino Camp and Obongi fishing villages in 2005. Percentage comparison of periportal fibrosis at Rhino Camp and Obongi in northern Ugandan. Periportal fibrosis in Rhino Camp ○ Periportal fibrosis in Obongi ▪

A total of 1,562 people from Rhino Camp and Obongi fishing villages along the Albert Nile in Northern Uganda were examined parasitologically for *S.mansoni* infection. These were from Rhino Camp, seven hundred and thirty three (n=733) 46.9% and Obongi (n=829) 53.1%. A total of (n=1273) 81.5% had *S. mansoni* eggs in their stools and (n=289) 18.5% were negative for *S.mansoni* infection in the field. The 289 negative specimens were further processed in the laboratory in Borne Germany using formal either concentration technique. Seven of them were positive with low egg count of an average of two eggs per low power microscopic field. Therefore, the true prevalence of *S.mansoni* infection is higher than 81.5% recorded in the field. The intensity of infection was classified as follows: Low 1–99epg, medium 100–499epg and high ≥500 epg (Sleigh et al. 1958). The distribution of different PFs in the different egg intensity in the Rhino Camp and Obongi fishing villages is shown in Figure 1. Age related intensity and the distribution in different grades of PF is shown in Figure 2. The distribution of PFs according to sex in the study patients was similar but their occupational activities were shown to influence the prevalence and the intensities of infections and consequentially the pathologies of the disease (Figure 3). The patterns of percentage of people with PFs in Rhino Camp and Obongi were similar in both 2005 and 2006 (Figure 4).

Percentage of reversibility of Pfs of people who had Abdominal ultrasound done on them in both years indicated that out of 506 patients found with no pathology in their livers in 2005 (Pf= 0); 395 (78%) remained at the same level. However, 98 (19%) of them developed light liver parenchyma changes, Pf grade (I). A small number of the patients, 13 (3%) presented moderate Pf grade (II). Two hundred fifty nine (259) patients showed Pf grade (I) in 2005 out of whom 169 (65%) became Pf (0) in 2006. Seventy four (23.6%) remained in Pf grade (I) and 16 (6.2%) digressed to Pf grade (II).

Out of the 147 (86%) patients with Pf grade (II) in 2005, 90 (61%) ameliorated their liver parenchyma damage to Pf grade (0) and 45 (30.6%) had light changes of their liver parenchyma to Pf grade (I), 6 (4.1%) remained in Pf grade (II) and another 6 (4.1%) of them digress to Pf grade (III). Among the 27 severe Pf grade (III) detected in the previous year, 8 (29.6%) reversed to Pf grade (0), another 8 (29.6%) improved to Pf grade (II) and 7 (25.9%) reverted to Pf grade (I). But 4 (14.8%) remained in Pf grade (III)

In general, in 2006, after a single dose treatment with Praziquantel 40mg/kg body weight, there were more people who were negative for *S.mansoni* infections (n=939) 73.8% unlike in the previous year where only (n=128) 18.5% had no *S. manson* in their stool. There were also improvement in the sonomorphological abnormalities of periportal fibrosis and organomorphometry of livers and spleens. In 2005, there were 433 patients with various grades of Pfs (I to III) versus 277 patients in 2006, a cure proportion of 156 (64%). Even of more significant one in 2005 grade (II) and grade (III) were observed in 174 patients but only 53 one year later, cure proportion of 121 (70%). Those 53 patients with grade (II) and grade (III) of liver involvement showed deterioration in PFs after treatment with praziquantel 40mg/kg body weight while living in the same environment.

## Discussion

According to the data presented, a positive influence of treatment with praziquantel 40mg/kg body weight on reversibility of liver PF changes due to *S.mansoni* infections in West Nile Uganda was observed. These observations agree with earlier works (Houston et al., 1993; Doehring-Schwedtfeger et al., 1992; Homeida et al., 1988). The positive trend was found in both young and old patients, with fibrosis of the liver. A similar finding was made by Homeida et al. (1988). These were more pronounced in patients with light liver PFs grade (I) and grade (II) as was earlier noted by Katz, et al. (1994). But patients with severe liver PFs grade (III) showed low reversibility of PFs. Since West Nile in Uganda is a hyper-endemic area for *S. mansoni*, this finding indicate that to sustain the reversibility of PFs, half yearly therapy with praziquaantel 40mg/kg body weight to the infected patients would prevent the development of severe PFs of the liver and encouraged reversibility of the lower PF grades. This phenomenon of sustainability of reversibility using repeated treatments has been demonstrated in previous studies (Homeida et al., 1988; Doerhing Schwerdtfeger et al., 1990; Abdel- Wahab et al., 1990).

Lakwo et al. (1994) in the same area of study showed that the water contact activities varied among the school children, fishermen and women but the overall frequencies of getting in contact with the water body at the Nile were similar. These different water contact activities included, playing and swimming in the water, washing utensils and clothing, collecting papyrus rids for making mats and fishing. These frequent water contact activities allow reinfection to take place. This could explain the irreversibility and deterioration of some of the PFs observed in this study. Because of ethical reason, there was no untreated group of patients set parallel to the study population to demonstrate natural PFs reversibility phenomenon. Jenkins et al., editor (1992) showed that beside careful evaluation of confounding factors such as improvement on the standard of living, changes of habits like water contamination with human excreta which encourage transmission of *S.mansoni* due to institutionalisation of health education, that is provision safe water and sanitation, also needed to be followed to ascertain their involvement in the reversibility of the liver damage in various grades of PFs. Mass chemotherapy treatment with antischistosomal drug is not considered as the only solution for those living in hyper-endemic areas for schistosome, but these data suggest that the life threatening complication of *S. mansoni*, liver fibrosis and consecutive portal hypertension easily observed with portable ultrasound machine, have been positively reduced one year after treatment with praziquantel as was also demonstrated earlier elsewhere (Homeida et al., 1988; Doerhing Schwerdtfeger et al., 1990; Abdel- Wahab et al., 1990).
